# Painful tumors in a patient with neurofibromatosis type 1: a case report

**DOI:** 10.1186/s13256-018-1847-0

**Published:** 2018-10-19

**Authors:** Niema Aqil, Salim Gallouj, Kaoutar Moustaide, Fatima Zahra Mernissi

**Affiliations:** Department of Dermatology, Hassan II University Hospital Center, Fes, Morocco

**Keywords:** Glomus Tumor, Neurofibromatosis 1, Finger, Subungual, Pain

## Abstract

**Background:**

Herein, we report an unusual case of multifocal glomus tumors in the same hand in a patient suffering from neurofibromatosis type 1.

**Case presentation:**

The patient was a 37-year-old Moroccan woman, suffering from neurofibromatosis type 1, with intense pain in the fingers, successfully treated with the excision of the tumors. Histology of the lesions confirmed the diagnosis of glomus tumor.

**Conclusion:**

We present this case to support the association between glomus tumors and neurofibromatosis type 1. Thus, we strongly recommend that one should suspect a glomus tumor in patients with neurofibromatosis type 1 if such patients have symptoms from finger pulp or nails.

## Background

Neurofibromatosis type 1 (NF1), also known as Von Recklinghausen’s disease, is an autosomal dominant disorder, with an incidence of 1 in 2500–3000 births and a prevalence of approximately 1 in 4000–5000 individuals [1]. It is caused by mutations in the NF1 tumor suppressor gene, located on chromosome 17 (17q11.2), which encodes neurofibromin, a protein able to downregulate the Ras-Raf/MAPK signaling pathway that activates cell proliferation [2]. Mutations of the NF1 gene result in alteration or loss of function of the negative regulator of growth and cellular differentiation of neurofibromin, leading to uncontrolled cell proliferation and increased risk of developing cancer [3, 4].

Glomus tumors are rare, accounting for less than 2% of all hand tumors. Usually occurring in adults in the fourth and fifth decades of life, these tumors originate from the glomus body, which is a modified neuron of neural crest origin. NF1 is associated with an increased risk of developing both benign and malignant tumors, especially those of neural crest origin. However, the association of glomus tumors of the fingers with NF1 is rarely observed [5].

In 1938, Klaber *et al*. [6] reported the first case of glomus tumors in patients with NF1.

Herein, we report on a case of a subungual glomus tumor associated with a glomus tumor glomangiomyoma type of the finger in a patient with NF1.

## Case presentation

**Fig. 1:**
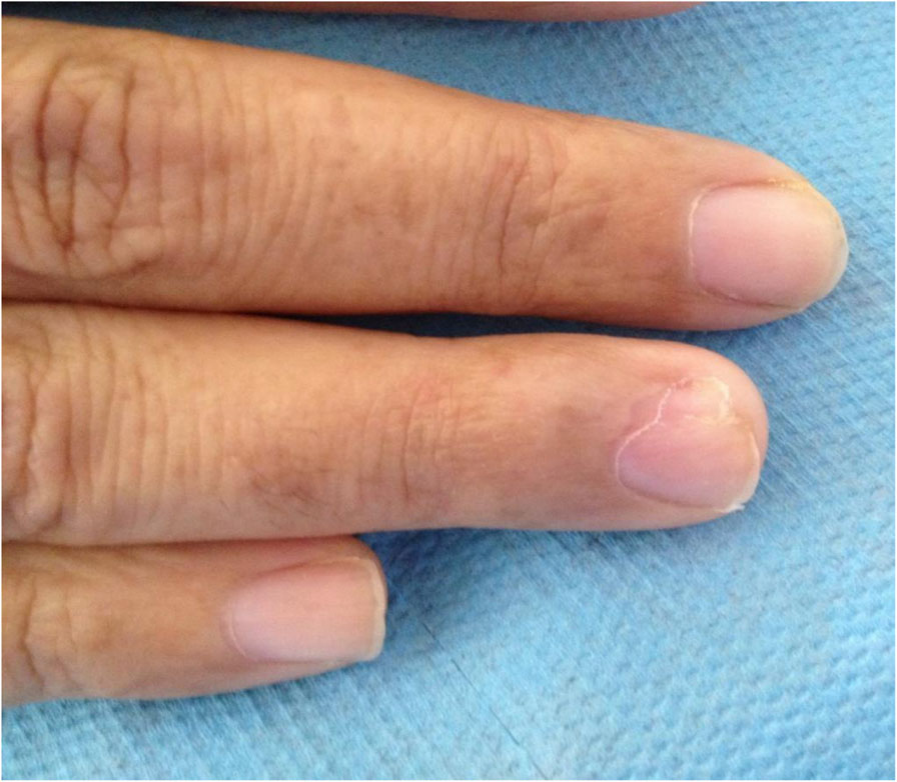
Nail deformation due to painful subungual tumor

**Fig. 2:**
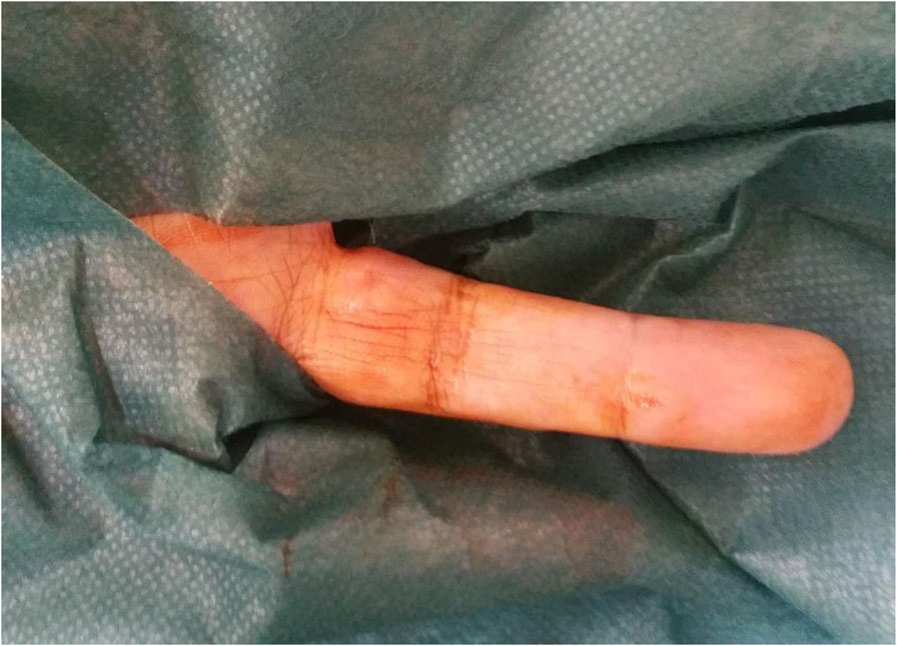
Painful subcutaneous tumor of the finger

A 37-year-old Moroccan patient, housewife, with treated latent syphilis, a non-smoker or alcohol drinker, diagnosed with NF1 on the basis of clinical features (more than six cutaneous coffee-milk spots, multiple neurofibromas, axillary lentigines, and mother as well as the sister and brother with NF1), presented with paroxysmal pain following exposure to cold or pressure on the fourth right subungual finger and in the palmar face of the first phalanx of the fourth right finger, for which she was taking a first-level analgesic. Clinically, the nail and its bed were almost normal; examination showed two painful subcutaneous tumors of approximately 0.5 cm in both locations, with gentle examination and palpation of the two tumors eliciting paroxysmal pain that did not allow a careful examination (Figs. 1 and 2).

**Fig. 3:**
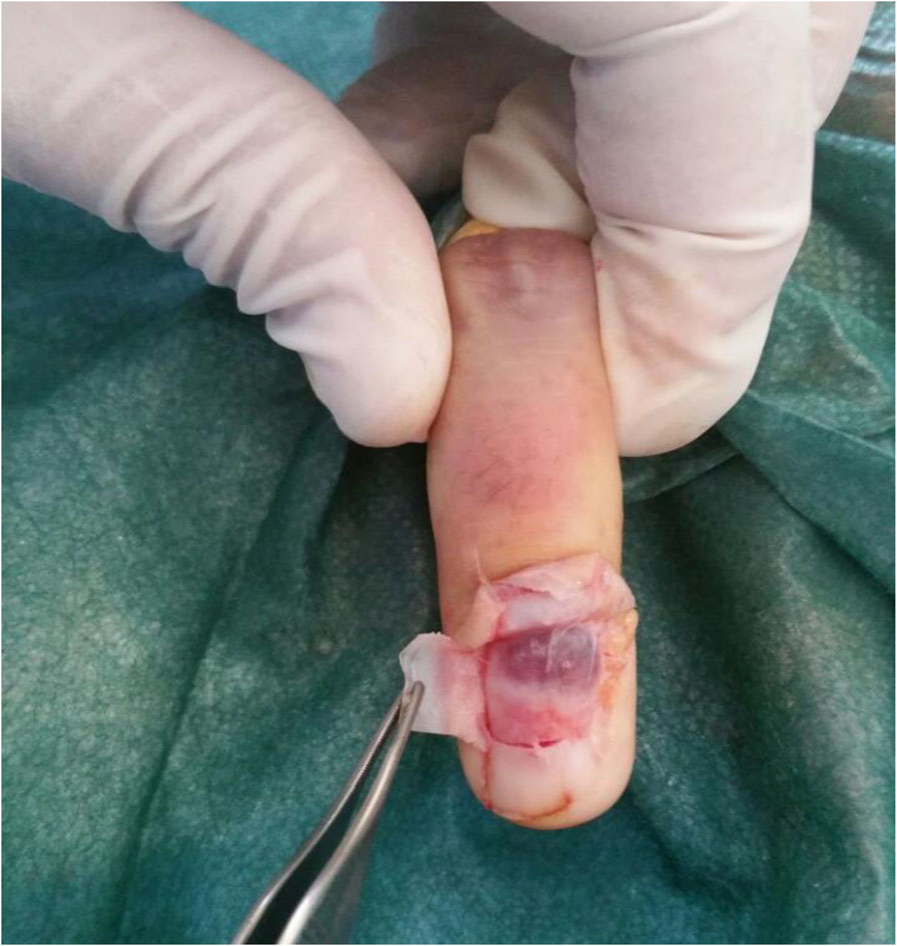
Intraoperative image showing a red-blue subungual tumor

**Fig. 4:**
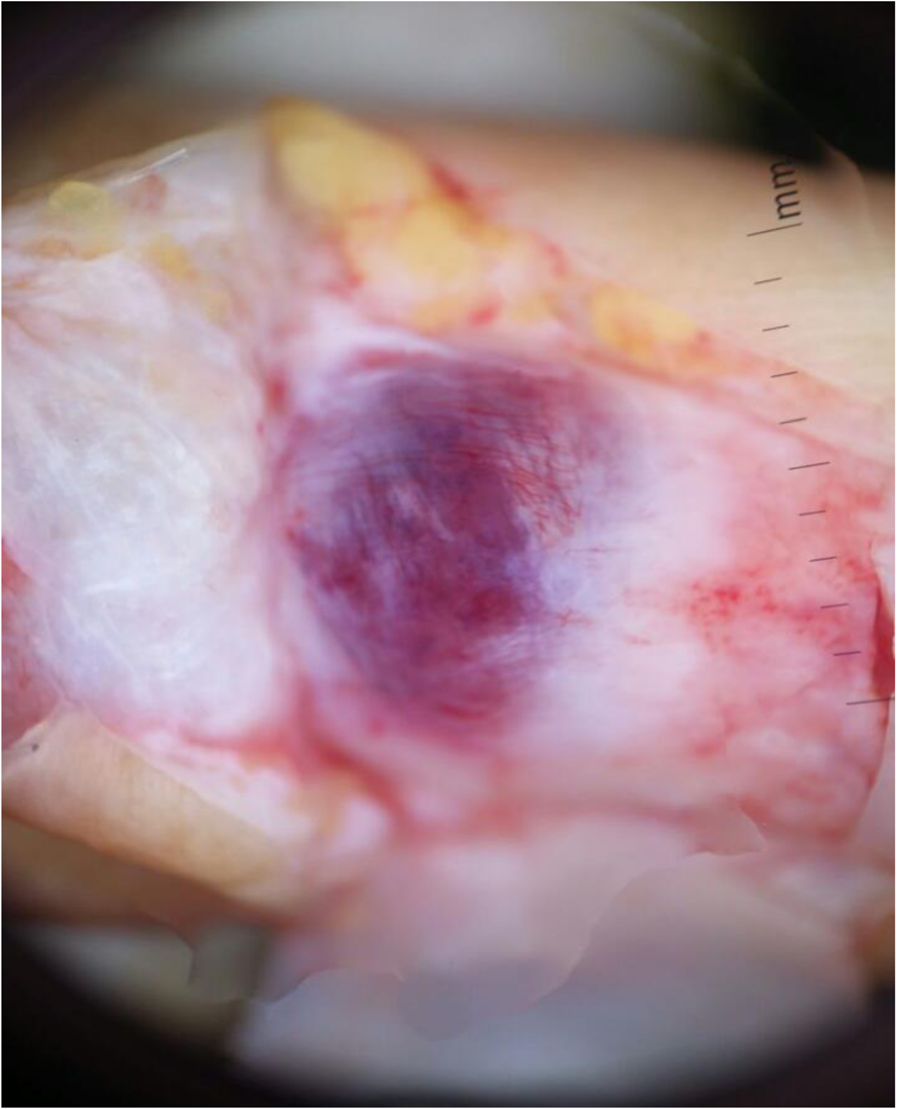
Intraoperative dermoscopy of the tumor of Fig. 1 showing a purplish-red area

**Fig. 5:**
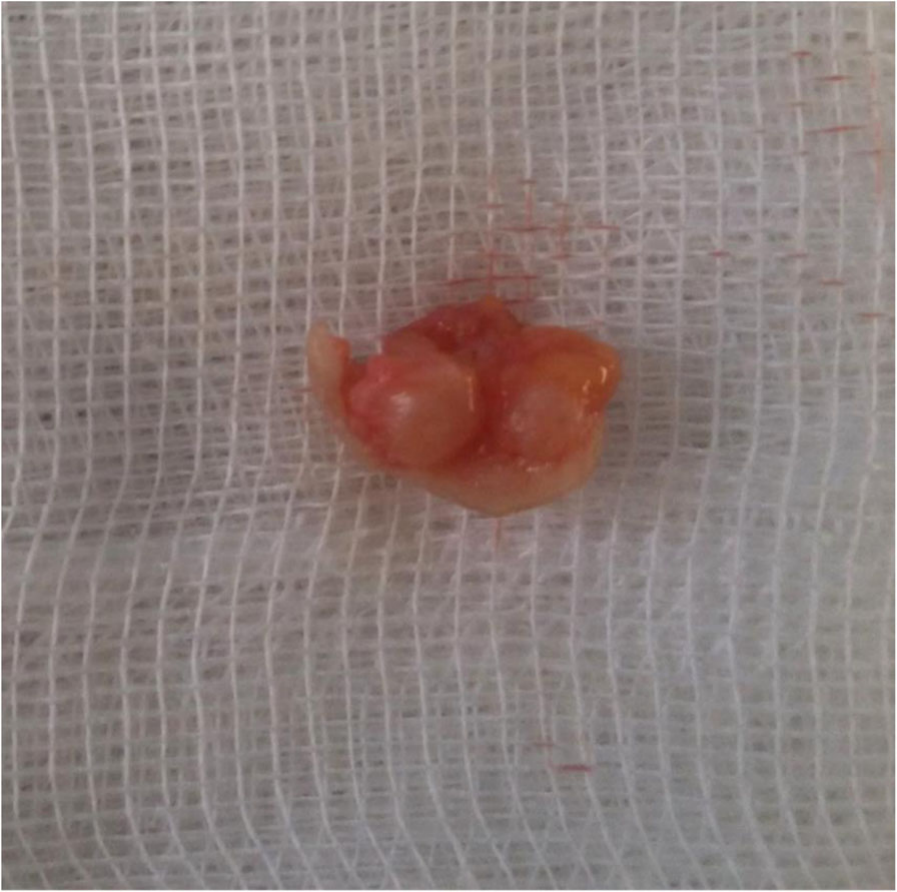
Image of the subcutaneous tumor of Fig. 2 after excision

**Fig. 6:**
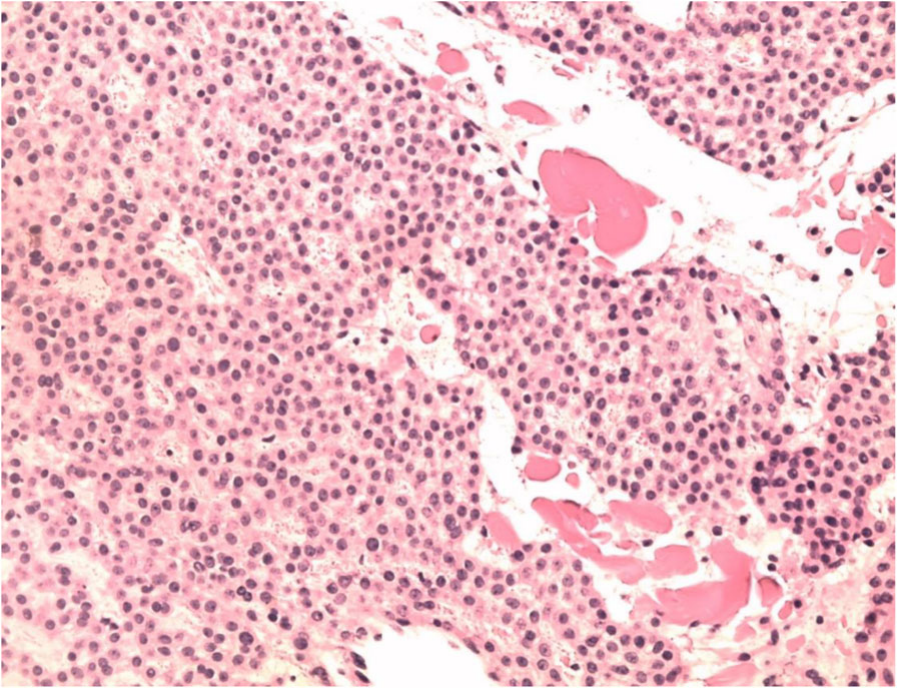
Histology of the subungual tumor: HES staining - > G × 200: Details of glomus cells: regular round cells

**Fig. 7:**
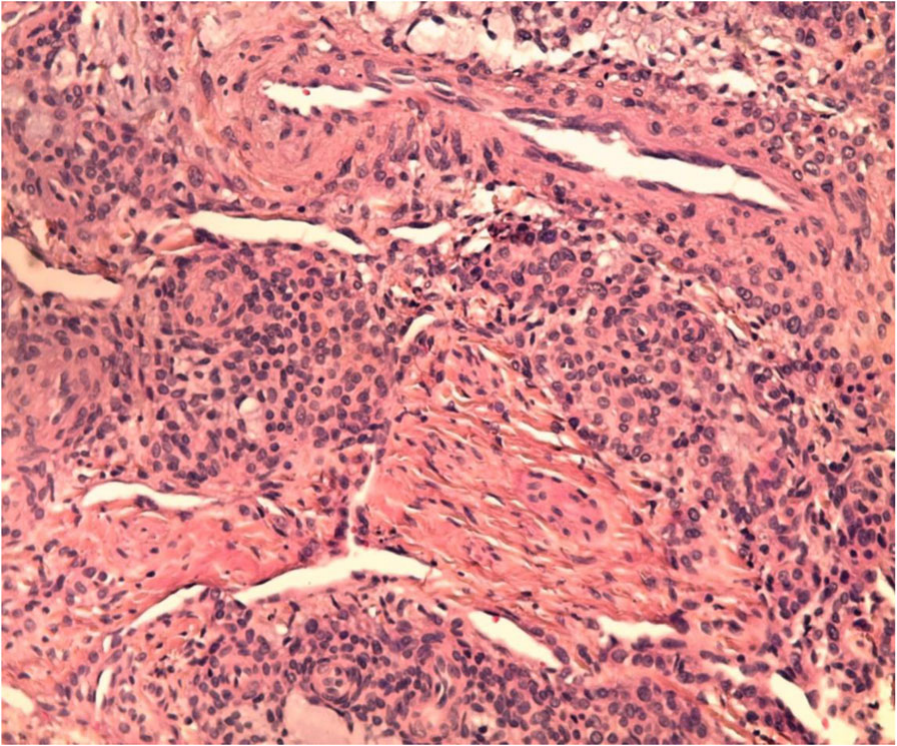
Histology of the subcutaneous tumor: HES staining - > G × 200: Glomangiomyoma with proliferation of smooth muscle cells

Following block anesthesia of the finger, an oblique incision was made at the level of the proximal fold of the nail. With partial elevation of the nail plate, the subungual tumor had a red-blue color (Fig. 3). Perioperative dermoscopy showed a purplish-red area reminiscent of a glomus tumor and elucidated the limits of extension of the tumor. Dermoscopy also guided the excision, which was made by removing the tumor encapsulated under the matrix of the nail; the latter was sutured after complete removal of the lesion (Fig. 4). The second tumor on the left hand was yellow-buff, suggestive of a schwannoma (Fig. 5). The histological examination of the first tumor confirmed the diagnosis of glomus tumor (Fig. 6), whereas the second was diagnosed as a glomangiomyoma-type glomus tumor (Fig. 7). The tumor cells were arranged around numerous narrow vascular slits that circumscribed flattened endothelial cells. These vessels were surrounded by several superimposed layers of ovoid cells with round, regular nuclei and moderately acidophilic cytoplasm. In some places, these elements deviated from the vascular walls and spread irregularly, sometimes isolated or in small clusters within a fibrous stroma, edematous, or myxoid. The histological examination of the second tumor eliminated the diagnosis of schwannoma and showed morphological aspects substantially comparable to the first tumor, with some peculiarities, including the presence of a fibrohyalin stroma and especially abundant myxoid. In some areas, the drawn vessels with irregular cavities were surrounded by thick smooth muscle without surrounding cell proliferation. In the superficial and middle dermis, a proliferation of glomic cells was observed around the vessels. These anatomopathological aspects were compatible with glomangiomyoma glomus tumor. Postoperative control showed a dramatic improvement in pain. The postoperative follow-up for 2 years was uneventful and the symptoms disappeared completely without recurrence and without deformation of the nail.

## Discussion

The present case, with two glomus tumors successfully excised, indicates, together with a few previously reported cases, that there is an association between glomus tumors and NF1. Glomus tumors in the hand are rare, and probably account for less than 2% of all hand tumors, predominantly occurring in middle-aged patients. The first case report indicating an association with glomus tumor and von Recklinghausen’s disease was published in 1937 [6], but few cases have been reported since [6–9].

The association is not well-known. Based on previous reports that patients with NF1 may have an increased incidence of glomus tumors, our working hypothesis was that this was such a tumor when the patient was referred. The suspicion that glomus tumors may be associated with NF1 is based mainly on a recent report of 11 patients with 20 glomus tumors in the fingers, and one in the toe [10]. Five of these patients had multiple tumors whereas our patient had two.

Interestingly, a meticulous analysis of these tumors showed that loss of neurofibromin function may be crucial in the pathogenesis of glomus tumors in NF1. Neurofibromin, which is a protein product of NF1 (tumor suppression gene NF1), regulates RAS through its GTPase (guanosine triphosphate) activity protein-related domain [10]. RAS mitogen-activated protein kinase (MAPK) hyperactivity was found in cultured glomus cells which lack NF1. The cells from the glomus tumors in the report by Brems *et al.* [10] showed increased activation of extracellular-regulated kinases 1 and 2 (ERK1/2) phosphorylation (p-ERK1/2) after stimulation with acidic fibroblast growth factor in the NF1-associated glomus tumor-derived glomus cells. An increased p-ERK:ERK ratio was therefore detected. Taken together, the data indicate an effect of NF1 inactivation on the MAPK pathway in NF1-associated glomus tumor-derived glomus cells. p-ERK1/2 is a prerequisite for proliferation of Schwann cells after damage to nerves in rats [11].

Most glomus tumors are solitary. Few cases of synchronous glomus tumors on the same digit [12] or in adjacent digits [13] have been reported. It is highly unlikely to have synchronous tumors in adjacent digits, as in the present case. However, in a patient with NF1, the risk of glomus tumors is increased [14]. As illustrated in this case, the diagnosis of multifocal glomus tumors should be considered in such patients, especially when the classic triad of clinical features (paroxysmal spontaneous pain, point tenderness, and cold hypersensitivity) is present. The majority are located in the subungual area. Delayed diagnosis and treatment may lead to unnecessary debilitation in such patients.

## Conclusion

The case report on herein provides further support for the notion that NF1 has an associated risk for multiple glomus tumors. For dermatologists managing NF1 patients, awareness of this association may facilitate early diagnosis and appropriate treatment. In particular, the symptoms of the patient and the perioperative dermoscopy seem to be useful in reaching a correct diagnosis.
